# Plant responses to high temperature and drought: A bibliometrics analysis

**DOI:** 10.3389/fpls.2022.1052660

**Published:** 2022-11-09

**Authors:** Yong Cui, Shengnan Ouyang, Yongju Zhao, Liehua Tie, Changchang Shao, Honglang Duan

**Affiliations:** Institute for Forest Resources and Environment of Guizhou, Key Laboratory of Forest Cultivation in Plateau Mountain of Guizhou Province, College of Forestry, Guizhou University, Guiyang, China

**Keywords:** bibliometric analysis, high temperature stress, drought stress, plant responses, yield, plant

## Abstract

Global climate change is expected to further increase the frequency and severity of extreme events, such as high temperature/heat waves as well as drought in the future. Thus, how plant responds to high temperature and drought has become a key research topic. In this study, we extracted data from Web of Science Core Collections database, and synthesized plant responses to high temperature and drought based on bibliometric methods using software of R and VOSviewer. The results showed that a stabilized increasing trend of the publications (1199 papers) was found during the period of 2008 to 2014, and then showed a rapid increase (2583 papers) from year 2015 to 2021. Secondly, the top five dominant research fields of plant responses to high temperature and drought were Plant Science, Agroforestry Science, Environmental Science, Biochemistry, and Molecular Biology, respectively. The largest amount of published article has been found in the Frontiers in Plant Science journal, which has the highest global total citations and H-index. We also found that the journal of Plant Physiology has the highest local citations. From the most cited papers and references, the most important research focus was the improvement of crop yield and vegetation stress resistance. Furthermore, “drought” has been the most prominent keyword over the last 14 years, and more attention has been paid to “climate change” over the last 5 years. Under future climate change, how to regulate growth and development of food crops subjected to high temperature and drought stress may become a hotspot, and increasing research is critical to provide more insights into plant responses to high temperature and drought by linking plant above-below ground components. To summarize, this research will contribute to a comprehensive understanding of the past, present, and future research on plant responses to high temperature and drought.

## Introduction

Global average surface temperatures have risen by 1.1°C since the Industrial Revolution and are expected to rise by 2-5°C by the end of the 21st century (Allan et al., 2021). The increase in average temperatures will lead to extreme meteorological events, and the frequency and intensity of extreme weather events (e.g., high temperatures and droughts) in most land areas may continue to increase in the future (Allan et al., 2021). The severity, duration, and frequency of extreme climates are likely to impact plant growth, physiology, and survival, leading to changes in vegetation structure and functioning (Allen et al., 2010; Medvigy et al., 2010; Siebers et al., 2015). Therefore, global scholars have conducted extensive research in related fields in recent years regarding the crucial potential impact of extreme weather events on plant individuals and ecosystems.

Many studies have shown that both high temperature and drought stress adversely affect plant growth and physiology (Din et al., 2015; Poudyal et al., 2019; Ro et al., 2021; Chen et al., 2022). High temperature stress can lead to stomatal closure through higher vapor pressure deficit (VPD), thereby limiting the supply of carbon dioxide (CO_2_) (Mathur et al., 2014). It may also impair photosynthetic apparatus and reduce the activity of photochemistry, resulting in decreased photosynthetic rate and associated physiological responses (e.g. reduced chlorophyll content and increased proline concentration) (Bhandari et al., 2018; Djanaguiraman et al., 2018; Rajametov et al., 2021; Thompson et al., 2022). Hence, high temperature often negatively affects plant growth and development (Hatfield and Prueger, 2015; Ogunkanmi et al., 2022). Meanwhile, drought stress can lead to the loss of cell turgor and lower water content (Hernandez-Santana et al., 2021), therefore limiting growth and dry mass accumulation (Ogunkanmi et al., 2022; Wan et al., 2022). Alternatively, plants often close stomata to limit water loss at the expense of photosynthesis (Yang et al., 2021). Under climate change, high temperatures and drought are often highly correlated (Ruehr et al., 2016). The extreme high temperature can accelerate soil evaporation, and increase plant transpiration *via* inducing the opening of stomata, thus aggravating the degree of drought stress (Berard et al., 2015). High temperature and drought in combination was observed to reduce photosynthetic rate and PSII photochemical efficiency to a greater extent than individual stress (Li et al., 2014; Wang et al., 2015; Ohnishi et al., 2019; Luo et al., 2021). The combined stress can also have significant impacts on grain yield and quality (such as Xerothermic wind disaster in wheat) and cause root self-thinning and transition into a state of dormancy in some tree species (Hlavacova et al., 2018; Ogunkanmi et al., 2022). Therefore, it is crucial to study the responses of plants to high temperature and drought.

Although a large number of research papers have described the effects of high temperature and drought stress on plants, these papers were limited by the time of publication, research institutions, and individuals, and were collected by different databases. Thus, it lacks of general synthesis of the effective information in specific subjects, and it is difficult to put forward the development trends and ideas in the field (Wallin et al., 2005; Zupic and Čater, 2015). Therefore, it is urgent to conduct a quantitative analysis of the literature to comprehensively summarize the research progress and trends in plant responses to high temperature and drought.

Bibliometric analysis is a modern way of assessing research based on fundamental bibliometric theory. It uses statistical mathematics to analyze, describe, and visualize literature in related research areas (Choudhri et al., 2015). The method can provide a description of existing knowledge states and new perspectives on characteristics and predict research trends on specific topics (Durieux and Gevenois, 2010; Zupic and Čater, 2015; He et al., 2022; Huang et al., 2022). In short, it can help researchers and policymakers quickly access the basics of the field and spot trends. The main contents of bibliometric analysis include (1) qualitative and quantitative analysis of publications indexed in databases based on statistical and computational techniques, (2) collaboration between different journals, countries and institutions, co-authors and co-occurrence categories, and (3) keywords for tracking and dynamic analysis. This analytical method has been widely used in literature analysis of information science, environmental science, agriculture, microbiology, geography and other disciplines (Andreo-Martinez et al., 2020; Romanelli et al., 2021; Wang et al., 2021; Castañeda et al., 2022).

To provide a systematic and objective insight into the development of scientific research on plant responses to high temperature and drought, this paper identified the bibliometric characteristics and visualized the relationship among papers in this field published in the Web of Science Core Collection database, hoping to improve understanding of the trends and prospects of research. The objectives of this study were to (1) sort out the research directions and priorities, and understand the historical development of plant responses to high temperature and drought research; (2) evaluate individual, institutional, and country contributions to research on plant responses to high temperature and drought; and (3) explore the change of research topics over time, and then predict future research directions for plant responses to high temperature and drought.

## Materials and methods

**Figure 1:**
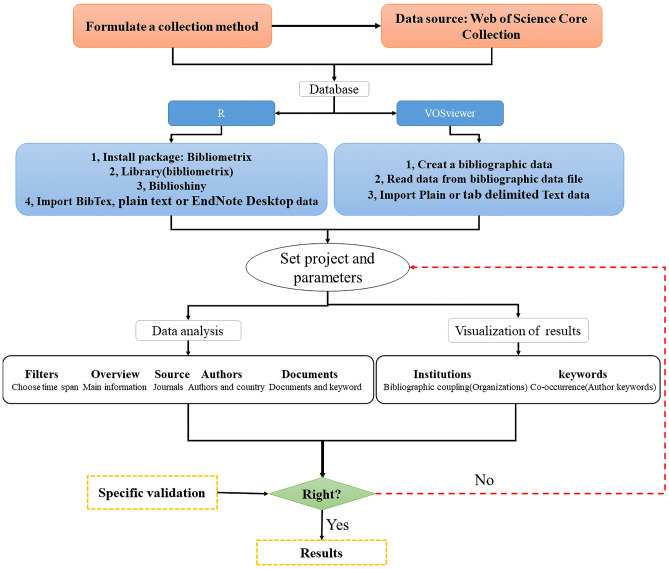
R & VOSviewer bibliometric analysis flowchart. This flowchart is modified from Huang et al. (2020).

Bibliometric analysis requires an extensive collection of databases on the topic of studies on the effects of high temperature and drought stress on plants. We used the Web of Science Core Collections from the database of Web of Science (WOS) (https://www.webofscience.com/WOS) to construct a bibliographic database on the topic of plant responses to high temperature and drought studies. WOS is broadly accepted by scholars and has been used as a helpful tool to do the bibliometric analysis in most recent research (e.g. Huang et al., 2020; He et al., 2022; Huang et al., 2022). Specifically, according to the “Advanced Search” option in WOS, we searched fields and information based on Boolean operators, using the terms of “high temperature stress” AND “drought stress”, and conducted a literature search from January 1, 2008 to December 31, 2021. The search was carried out for “All Fields” in Web of Science Core Collection. Furthermore, we improved the accuracy of keywords through the “Exact Search” option (The “Advanced Search” policy can be obtained from the Science Web All Databases Help or http://images.webofknowledge.com//works535r111/Help/WOK/HP_Advanced_Search, and the “Exact Search” can be chosen from “Advanced Search” interface.). Finally, we utilized manual filtering of search results to remove irrelevant articles and then analyzed results by R and VOSviewer (**Figure 1**).

### R-based “bibliometrix” package

A bibliometric tool (or “bibliometrix” package in R) was developed, following the logical workflow of classical bibliometrics (Aria and Cuccurullo, 2017) and combining statistical calculations and visualizations. The “bibliometrix” software package makes the most of the statistical and tracing capabilities of the R language. In addition, R is also an object-oriented, functional programming language, which means that “bibliometrix” packages may be combined with other packages to create new functions. After obtaining the database about high temperature stress and drought stress in WOS studies (saved as BibTex or plain text or EndNote Desktop), we performed a bibliometric analysis of the scientific research on plant responses to high temperature and drought step by step (**Figure 1**).

### VOSviewer

VOSviewer is a literature analysis and knowledge visualization software developed by Leiden University in the Netherlands (van Eck and Waltman, 2010). It is a free bibliometric visualization tool that can effectively view research problems through clustering views, label views, and density views, and has distinct advantages in analyzing Keyword analysis, cluster analysis, subject words, author information, and other aspects. After generating the database of high temperature stress and drought stress research in WOS (saved as plain text or tab-delimited), we followed the steps to conduct a bibliometric analysis (**Figure 1**).

## Results

### The principal information about collected papers

**Figure 2:**
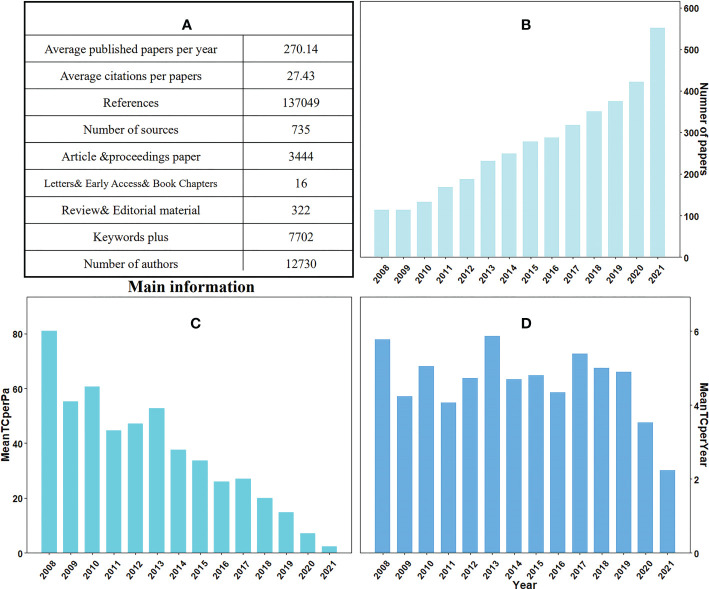
Information based on 2008-2021 studies on plant responses to high temperature and drought, including **(A)** additional information about plant responses to high temperature and drought in the table; **(B)** number of papers in 2008-2021; **(C)** the average total number of citations per paper from 2008 to 2021 (MeanTCperPa) and **(D)** average total number of citations per year (MeanTCperYear).

From 2008-2021, a total of 3782 papers on plant responses to high temperature and drought were published in 735 sources, including 88.4% articles, 8.4% review papers, 2.7% conference proceedings, 0.3% book chapters, 0.1% editorial materials, 2 letters and 1 retracted publication (**Figure 2A**). We also found that 90.4% of the papers were published in recent 10 years. The number of papers can reflect the popularity of the research field to a certain extent. In 2008-2010, only 361 papers on plant responses to high temperature and drought were published, accounting for 9.4% of the total papers. The number of papers increased significantly between 2011 and 2015 (1116 papers, or 29.5% of the total), and the average number of papers increased from 181 (2008-2010) to 279 (2011-2015) per year. Between 2016 and 2021, the number of papers increased exponentially, with a total of 2294 published, accounting for approximately 60.6% of the total papers (**Figure 2B**). In the past 6 years, more papers have been published in this field than in the previous 8 years, indicating that scientific issues related to this field have attracted more and more attention from scientists and the society. For the citation of papers, the highest mean value of global total citations was 80.96 times in 2021, and it was the lowest in 2008 with only 2.23 times (**Figure 2C**).

In addition, interannual variations, such as the appearance of extreme weather conditions, can also affect the number of citations of the papers about the research of plant responses to high temperature and drought. We also calculated the average total number of citations per article and year. The highest mean total quote was in 2008 (5.78 times) and 2013 (5.86 times), compared with the lowest average total citation was only 2.23 times in 2021 (**Figure 2D**). Approximately 270 articles were published annually on plant responses to high temperature and drought. The total number of citations worldwide is 103741 times, with an average of 27.43 times per article and 5.73 times per document per year. Additionally, a total of 12730 authors was participated in the study of plant responses to high temperature and drought and contributed 8820 keywords at the same time (**Figure 2A**). Consequently, research on plant responses to high temperature and drought is receiving increasing attention from publishers, regions, institutions, and researchers.

### Publisher’s citations and sources

Regarding research into plant responses to high temperature and drought, most of the papers were published in the fields of plant science, molecular science, and environmental science. The top 5 journals that have published the most papers were Frontiers in Plant Science (192 papers), Plos One (83 papers), International Journal of Molecular Sciences (79 papers), Environmental & Experimental Botany (73 papers), and Journal of Experimental Botany (69 papers). The top 5 journals with global total citations were Frontiers in Plant Science (8074), Journal of Experimental Botany (6422), Plant Cell & Environment (3292), Plant Physiology (2659), Environmental & Experimental Botany (2383) (**Table 1**).

**Table 1:** The most relevant source information between 2008-2021 in papers relating to plant responses to high temperature and drought.

Rank	Sources	TC	TP	H-index	IF
1	Frontiers in Plant Science	8074	192	47	5.753
2	Journal of Experimental Botany	6422	69	39	6.992
3	Plant Cell & Environment	3292	39	30	7.228
4	Plant Physiology	2659	25	28	8.34
5	Environment & Experimental Botany	2383	73	27	5.545
6	Plos One	2138	83	24	3.24
7	International Journal of Molecular Sciences	2031	79	23	5.923
8	Bmc Genomics	1843	39	22	3.969
9	Bmc Plant Biology	1784	44	22	4.215
10	Photosynthetica	1664	29	22	3.189
11	New Phytologist	1658	19	21	10.151
12	Plant Physiology & Biochemistry	1653	68	21	4.27
13	Field Crops Research	1543	47	21	5.224
14	Plant Cell Reports	1502	31	21	4.57
15	Journal of Agronomy & Crop Science	1494	52	20	3.473
16	Global Change Biology	1483	24	19	10.863
17	Planta	1405	28	19	4.116
18	Tree Physiology	1295	37	19	4.196
19	Plant Molecular Biology	1253	29	18	4.076
20	Crop Science	1249	41	18	2.319

TC is total global citations; TP is total papers, and IF is the impact factor (2021).

**Figure 3:**
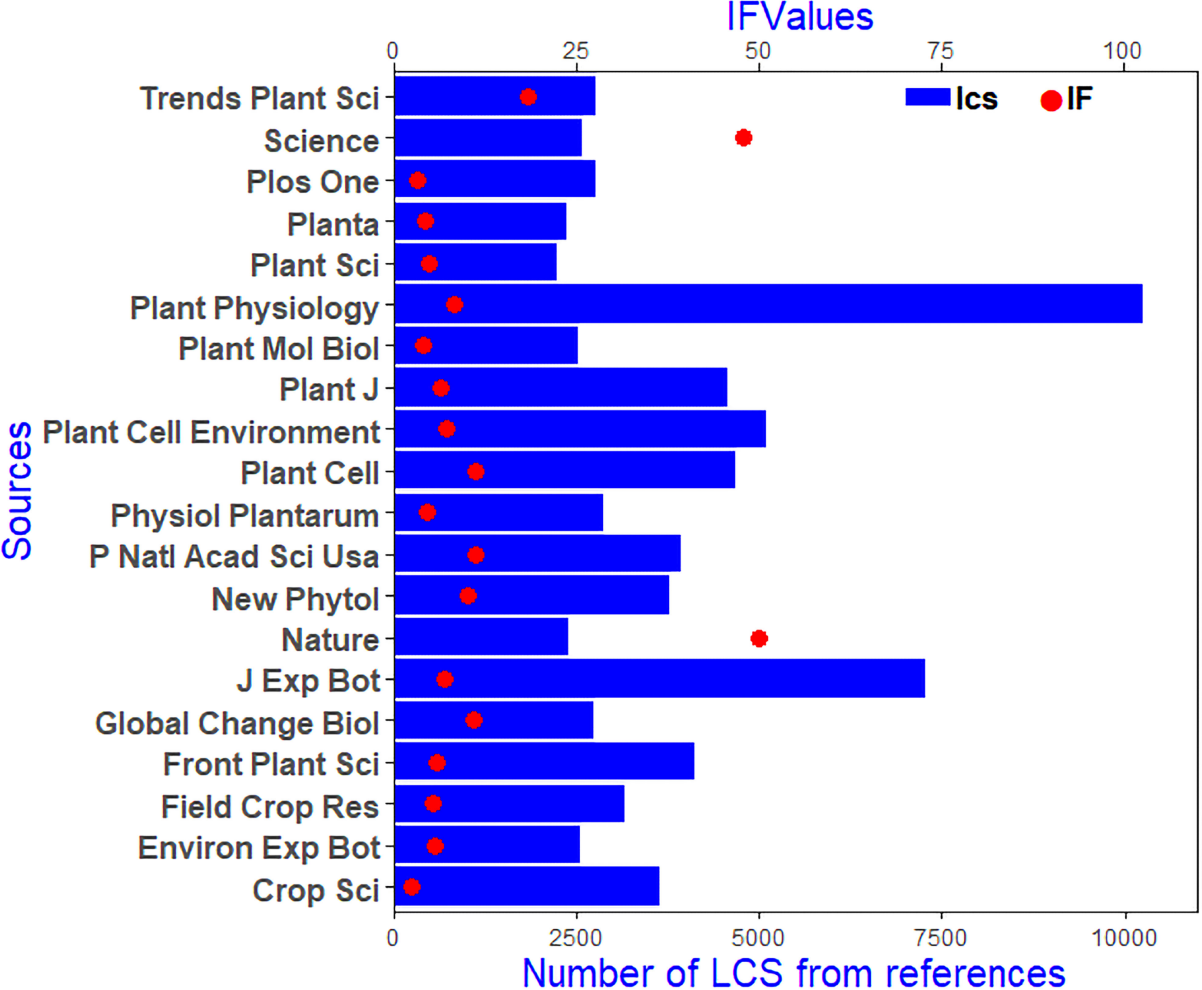
Most local cited sources (LCS) about the research of plant responses to high temperature and drought from 2008 to 2021. The blue histogram represents the number of LCS, and the red dot is the journal’s impact factor (2021).

The H index, which indicates that H articles have been cited at least H times, is generally used to assess the academic impact of a journal or individual. Thus, the H Index can represent journal’s or person’s academic achievements. Among all the journals related to plant responses to high temperature and drought research, the top 5 H-indexed journals were Frontiers in Plant Science (46), Journal of Experimental Botany (37), Plos One (29), Environmental & Experimental Botany (28), Plant Cell & Environment (27) (**Table 1**). For locally cited references for all papers, the top 5 journals were Plant Physiology (10175), Journal of Experimental Botany (7195), Plant Cell & Environment (5060), Plant Cell (4629), and Plant Journal (4516) (**Figure 3**). In general, the main research directions of research on plant responses to high temperature and drought were plant science, agroforestry science, environmental science, biochemistry, and molecular biology. Frontiers in Plant Science ranked first in terms of the number of papers, reference citations, and the H-index (**Table 1** and **Figure 3**).

### The top 10 countries for papers published and cited in different periods

The number of published papers and global citations in a country’s research can reflect the scientific research level and emphasis. Between 2008 and 2021, 117 nations studied plant response to high temperature and drought. The results showed that China had the largest number of published and cited papers (1230 papers, each paper was cited 28.52 times). In comparison, the United States ranked the second (702 papers, each paper was cited 33.27 times) and India the third (1199 papers, each paper was cited 33.49 times). In countries with more than 2000 citations, the average number of cited papers in the Austria reached the highest 59.54 times, and each paper had the highest number of citations during 2008-2011 and 2018-2021 (**Table 2**), indicating that it had high-quality papers in the field of plant responses to high temperature and drought. From 2008 to 2012, China (206 papers and 12440 citations) maintained the highest number of papers and citations, while the United States (122 papers and 7325 citations) and India (54 papers and 3632 citations) ranked the second and third, respectively. From 2013-2017, the number of papers published in China (429 papers) increased considerably from 2008-2012, and the total number of citations (14475) was also the highest of all relevant countries. However, Pakistan (67.40 citations per paper) was the country with the most citations per paper in 2013-2017 (**Table 2**). Between 2018 and 2021, the number of papers published in China grew steadily (595 papers), and it should be noted that the number of papers published in Pakistan (104 papers) has doubled in this period. In general, China, USA and India were the countries that contributed the most to plant responses to high temperature and drought research, which maintained a high level of total papers published, research impact, quality, and total citations in the three periods.

**Table 2:** Dynamic changes in the total number of papers and citations of the top 10 countries in the plant responses to high temperature and drought study from 2008-2012, 2013-2017, and 2018-2021.

Rank		Periods2008-2012			2013-2017			2018-2021		
	Nation	TP	TC	Nation	TP	TC	Nation	TP	TC
1	CHINA	206	12440	CHINA	429	14475	CHINA	595	8163
2	USA	122	7325	USA	256	10962	USA	324	5066
3	INDIA	54	3632	INDIA	151	7087	INDIA	196	2712
4	GERMANY	44	3752	GERMANY	104	4752	GERMANY	114	1906
5	JAPAN	37	2028	AUSTRALIA	81	4609	AUSTRALIA	111	2775
6	SPAIN	34	1796	SPAIN	68	3436	PAKISTAN	104	1844
7	KOREA	33	2237	ITALY	67	2428	SPAIN	98	1365
8	ITALY	33	2535	FRANCE	55	2633	ITALY	66	1364
9	CANADA	30	1867	PAKISTAN	52	3505	CANADA	55	938
10	AUSTRALIA	29	2251	KOREA	46	1237	BRAZIL	51	435

TP is the total number of papers, and TC is the total number of citations worldwide.

### Most influential contributors and institutions

**Figure 4:**
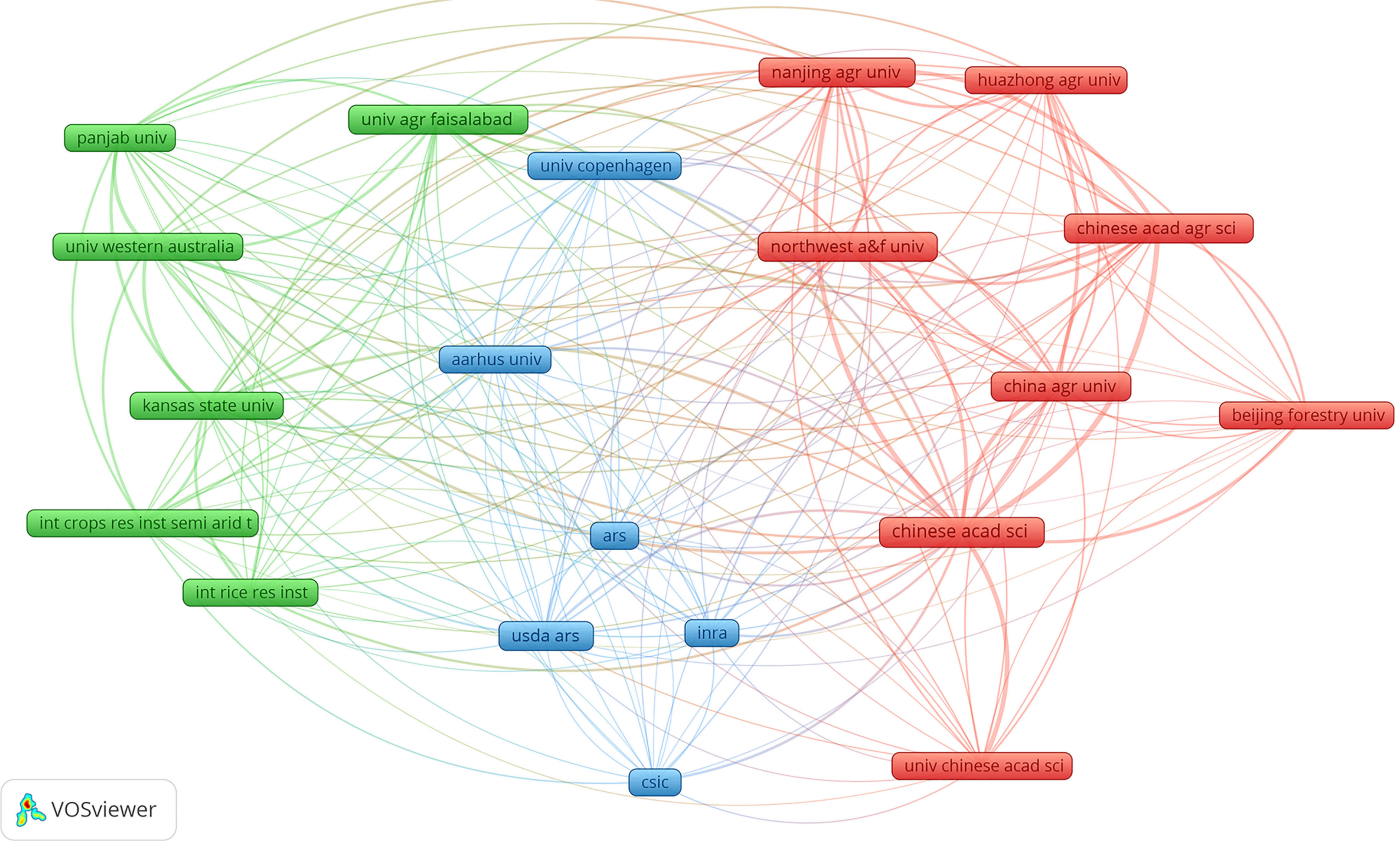
Cooperative network analysis of the top 20 production institutions or universities based on the VOS clustering algorithm. Nodes represent different institutions or universities. Different colors represent different clustering of results. Different colored connecting lines indicate the partnership between institutions or universities.

As scientific research develops, more and more studies are accomplished through mutual cooperation between different countries and institutions, which can produce more significant influence and scientific research value. The clustering of all research institutions was performed using the VOS clustering algorithm and then to select top 20 institutions that published articles to explore cooperative relationships between institutions (**Figure 4**). The cooperative network was mainly clustered into three categories represented by Northwest A&F University, Nanjing Agricultural University, and China Agricultural University. Northwest A&F University published the most papers (173 papers). These papers mainly focused on (a) the conserved structure of plant heat shock protein families, genome-wide identification-based plant heat shock protein gene families and their expression profiles in abiotic stress, and different regulatory levels and functions (Guo et al., 2016), (b) the MsZEP gene which may be involved in alfalfa responses to different abiotic stresses and nodules which can improve drought tolerance and salt tolerance of transgenic tobacco through heterozygous genes (Zhang et al., 2016), (c) endophytes that produce melatonin in grape roots promoting the production of host endogenous melatonin induced by abiotic stress (Jiao et al., 2016), (d) heat shock protein: a dynamic biomolecule that fights plant biology and abiotic stress (ul Haq et al., 2019), and (e) characteristics of rice NADPH oxidase gene and its expression under different environmental conditions (Wang et al., 2013). These cooperative institutions had many research directions on plant responses to high temperature and drought, but the main directions were (a) the regulation and expression of plant genes (Liao et al., 2008; Liu et al., 2008; Xue et al., 2009; Huang et al., 2010; Fang et al., 2015; Guo et al., 2016), (b) root responses (Dinneny et al., 2008; Yu et al., 2008), (c) plant signaling (Huang et al., 2012), and (d) crop yield and quality (Wang and Frei, 2011). From 2008 to 2021, 12730 authors were participated in studies related to high temperature and drought stress. The highest individual total citations (1553) and the most published papers (15 papers, H index = 13) and total local citations (189 times) were Jagadish Svk (**Table 3**) from Texas Tech University of USA, whose main research direction is how high temperatures affect wheat, rice, and other crops, including research on plant productivity, molecular mechanisms, metabolic regulation, and other topics (Wassmann et al., 2009; Jagadish et al., 2010; Rang et al., 2011; Prasad et al., 2017).

**Table 3:** The highest cited authors of studies in plant responses to high temperature and drought from 2008 to 2021, based on two statistical indicators (TC and LTC) and the number of papers published (TP).

Rank	Author/Institution	TC	LTC	TP	H-index
1	Jagadish Svk/Texas Tech Univ	1553	189	15	13
2	Zhang X/Univ of Tokyo & Northeast Forestry Univ	1546	37	62	22
3	Ashraf M/Univ of Agriculture Faisalabad	1405	54	12	8
4	Li X/Max-Planck-Institute of Molecular Plant Physiology	1402	56	52	20
5	Wang L/Univ of Tasmania	1391	42	44	20
6	Zhang Y/Institute of Tibetan Plateau Research	1271	52	64	22
7	Xu Z/State Key Laboratory of Vegetation and Environmental Change, Institute of Botany, Chinese Academy of Sciences	1254	29	16	10
8	Liu J/National Laboratory of Plant Molecular Genetics, Institute of Plant Physiology and Ecology	1222	56	36	15
9	Heuer S/MAN Truck & Bus SE	1217	170	7	7
10	Liu Y/Shandong Agricultural Univ	1216	66	49	18
11	Hasanuzzaman M/Sher-e-Bangla Agricultural Univ	1215	141	10	9
12	Wang X/Northeast Agricultural Univ	1188	39	59	22
13	Nahar K/Kagawa Univ	1182	136	9	8
14	Huang J/Nanjing Agricultural Univ	1178	98	14	9
15	Wu C/Huazhong Agricultural Univ	1175	71	9	5
16	Prasad Pvv/Kansas State Univ	1164	259	24	13
17	Li Y/Beijing Forestry Univ	1151	19	51	20
18	Fahad S/Huazhong Agricultural Univ	1138	88	9	9
19	Zhang L/Institute of Tropical Bioscience and Biotechnology	1138	62	42	17
20	Fujita M/Kagawa Univ	1127	135	9	8

TC is the total number of citations worldwide; LTC is the total local number of references; TP is the total number of papers.

### Most cited papers and references

Conventionally, the number of papers’ citations directly reflects the quality and influence of this paper globally or locally. This study compiled the top 20 cited papers on plant responses to high temperature and drought research (**Table 4**). The most totally cited paper was published in the Journal of Experimental Botany by Krasensky and Jonak (2012) (**Table 4**). The paper published by Barnabas et al. (2008) was the most locally totally cited in the journal of Plant, Cell and Environment during 2008-2021 (**Table 5**). It was necessary to classify papers according to the contents of the research. For physiological and ecological responses, it can be summarized in the following points: (a) Plant metabolism. Krasensky and Jonak (2012) summarized the information on metabolic regulation under drought, extreme temperature and salinity stress, and introduced the signaling events that mediate stress-induced metabolic changes. Yang et al. (2018) provided an overview of the environmental factors that contributed to fluctuations in plant secondary metabolite SMs, provided practical methods for obtaining consistent quality and high quantities of bioactive compounds in vegetation, and gave some recommendations for future research and development. (b) Yield. Barnabas et al. (2008) summarized the currently available information on grain propagation in the context of plant responses to high temperature and drought, with a new respective toward potential strategies to improve cereal yield safety. Bita and Gerats (2013) presented the latest research advances in abiotic stresses, reviewing possible procedures and methods, which could lead to the production of new varieties with sustainable yield production in a world challenged by higher average temperatures and greater temperature fluctuations. Nardone et al. (2010) reviewed the impact of climate change on livestock and projected an increase in the frequency of drought events around the world, which could directly affect pasture and crop production. Their findings are important to guide livestock development in future climate change. (c) Signal transduction. Huang et al. (2012) first elaborated on the general pathway of stress signal transduction, and then focused on various aspects of biological stress signal transduction networks and discussed common regulatory systems and crosstalk between biological stress, especially the MAPK cascade and the crosstalk between ABA signals and biological signals. Vishwakarma et al. (2017) elucidated the importance and role of ABA signaling in various stresses and the regulation of ABA biosynthetic pathways and stress tolerance transcription factors. Miura and Tada (2014) reviewed the effects of SA (salicylic acid) on regulating water stress response and stomatal closure. (d) Physiological changes and responses. Akram and Ashraf, (2013) conducted a detailed review of the changes in various gas exchange characteristics of different types of stress, especially in agricultural plants, and discussed the components of the current worldwide identification signal. Suzuki et al. (2014) provided the latest advances in research on plant responses to different stress combinations. In particular, they discussed how different stress responses were integrated and affected plant growth and physiological properties. Niinemets (2010) analyzed the physiological responses of trees to key environmental stress factors and their combinations from seedlings to mature trees. Xu and Zhou (2008) studied the photosynthesis of perennial *Leymus Chinensis* under different soil moisture conditions. Maurel et al. (2015) established the critical role of aquaporins in plant integrative biology. Hasanuzzaman et al. (2013) reviewed recent findings on responses, adaptations, and tolerance to high temperatures at the cellular, organ, and whole plant levels, and described various approaches to improve plant heat tolerance. Fahad et al. (2017) detailed the responses of plants to heat and drought stress, with particular emphasis on their commonalities and differences. They provided a comprehensive overview of traditional and modern methods, of coping with heat and drought stress and critical discussions on plant responses to these two abiotic stresses.

**Table 4:** The most cited papers from studies in plant responses to high temperature and drought from 2008 to 2021, based on two statistical indicators (TC and LTC).

Rank	Author	DOI	TC	LTC
1	(Krasensky and Jonak, 2012)	10.1093/jxb/err460	1100	84
2	(Barnabas et al., 2008)	10.1111/j.1365-3040.2007.01727.x	1009	215
3	(Ashraf and Harris, 2013)	10.1007/s11099-013-0021-6	949	44
4	(Bita and Gerats, 2013)	10.3389/fpls.2013.00273	774	115
5	(Suzuki et al., 2014)	10.1111/nph.12797	763	67
6	(Hasanuzzaman et al., 2013)	10.3390/ijms14059643	733	100
7	(Fahad et al., 2017)	10.3389/fpls.2017.01147	672	68
8	(Liu et al., 2008)	10.1261/rna.895308	627	14
9	(Hundertmark and Hincha, 2008)	10.1186/1471-2164-9-118	622	30
10	(Dinneny et al., 2008)	10.1126/science.1153795	493	4
11	(Lata and Prasad, 2011)	10.1093/jxb/err210	480	54
12	(Nardone et al., 2010)	10.1016/j.livsci.2010.02.011	479	4
13	(Jeong et al., 2010)	10.1104/pp.110.154773	461	31
14	(Huang et al., 2012)	10.1007/s11033-011-0823-1	458	25
15	(Vishwakarma et al., 2017)	10.3389/fpls.2017.00161	434	13
16	(Niinemets, 2010)	10.1016/j.foreco.2010.07.054	418	0
17	(Xu and Zhou, 2008)	10.1093/jxb/ern185	416	13
18	(Yang et al., 2018)	10.3390/molecules23040762	383	2
19	(Maurel et al., 2015)	10.1152/physrev.00008.2015	382	8
20	(Miura and Tada, 2014)	10.3389/fpls.2014.00004	352	10

DOI is the digital object unique identifier; TC is the total global number of references; LTC is the total local number of references.

**Table 5:** The most cited references for research papers on plant responses to high temperature and drought from 2008 to 2021.

Rank	Author	DOI	TC	LTC
1	(Livak and Schmittgen, 2001)	10.1006/METH.2001.1262	116987	256
2	(Liu et al., 1998)	10.1105/TPC.10.8.1391	2546	254
3	(Wahid et al., 2007)	10.1016/J.ENVEXPBOT.2007.05.011	2039	242
4	(Barnabas et al., 2008)	10.1111/J.1365-3040.2007.01727.X	1072	215
5	(Bradford, 1976)	10.1016/0003-2697(76)90527-3	234001	193
6	(Mittler, 2006)	10.1016/J.TPLANTS.2005.11.002	1624	191
7	(Rizhsky et al., 2004)	10.1104/PP.103.033431	1061	189
8	(Dubouzet et al., 2003)	10.1046/J.1365-313X.2003.01661.X	1350	184
9	(Bates et al., 1973)	10.1007/BF00018060	NA	174
10	(Yamaguchi-Shinozaki and Shinozaki, 1994)	10.1105/TPC.6.2.251	1729	171
11	(Stockinger et al., 1997)	10.1073/PNAS.94.3.1035	1528	148
12	(Yamaguchi-Shinozaki and Shinozaki, 2006)	10.1146/ANNUREV.ARPLANT.57.032905.105444	2081	145
13	(Thomashow, 1999)	10.1146/ANNUREV.ARPLANT.50.1.571	2611	143
14	(Clough and Bent, 1998)	10.1046/J.1365-313X.1998.00343.X	15235	138
15	(Mittler, 2002)	10.1016/S1360-1385(02)02312-9	8340	136
16	(Rizhsky et al., 2002)	10.1104/PP.006858	725	136
17	(Kasuga et al., 1999)	10.1038/7036	1736	134
18	(Allen et al., 2010)	10.1016/J.FORECO.2009.09.001	4335	133
19	(Sakuma et al., 2002)	10.1006/BBRC.2001.6299	1493	132
20	(Zhu, 2002)	10.1146/ANNUREV.ARPLANT.53.091401.143329	4279	120

DOI is the digital object unique identifier, TC is the total global number of references, and LTC is the total local number of references.

For molecular biological responses: Liu et al. (2008) found that there was considerable crosstalk between drought stress signaling pathways, expanding the current view that miRNAs are ubiquitous regulators under stressful conditions. Hundertmark and Hincha (2008) conducted a genome-wide analysis of the *Arabidopsis* LEA protein and its encoding genes expression using quantitative RT-PCR technology. The high proportion of retained repetitive genes and putative functional diversification suggests that they provided an evolutionary advantage for organisms under different stressful environmental conditions. This comprehensive analysis can be an important starting point for future efforts to elucidate the functional roles of these enigmatic proteins. Dinneny et al. (2008) investigated the transcriptional response to high salinity in different cell layers and developmental stages of *Arabidopsis* roots. They found that transcriptional responses were highly restricted by developmental parameters and known stress pathways primarily controlled semi-universal responses, revealing cell-layer-specific and interlayer effects by using mutants. Lata and Prasad (2011) reviewed recent research progress and focused on the role of the DRE binding family in adapting to different abiotic stress responses and its structural and functional characteristics, emphasizing the expression and regulation of DREB. The application value of DREBS in crop improvements, such as crop resistance engineering and molecular marker-assisted selection was discussed. Jeong et al. (2010) reported the results of a functional genomics approach that identified a rice NAC (NAM [apical meristem], ATAF1-2, and CUC2 [cup cotyledon]) domain gene OsNAC10, which improved the performance of transgenic rice under drought conditions in the field. The results showed that root-specific overexpression of OsNAC10 could expand roots and enhance the drought resistance of transgenic plants, thereby significantly increasing grain yield under drought conditions in the field.

Among the references, the papers with the highest TC and LTC are written by Livak and Schmittgen (2001) and mainly investigated the question by analyzing relative gene expression data using real-time quantitative PCR (**Table 5**). The focus of these references was also consistent with the research direction of plant responses to high temperature and drought in 2008-2021, all of which was to explore the effects of abiotic stress on plants from different perspectives of individual plants (**Table 5**). An exception is Allen et al. (2010), which was the first global assessment of recent 3 decades tree mortality due to drought and heat stress at the ecosystem level. These references provided essential insights into the relationship between high temperature, drought stress, and plant growth as well as the impacts of future climate change on plants.

### The development and co-occurrence of keywords

**Figure 5:**
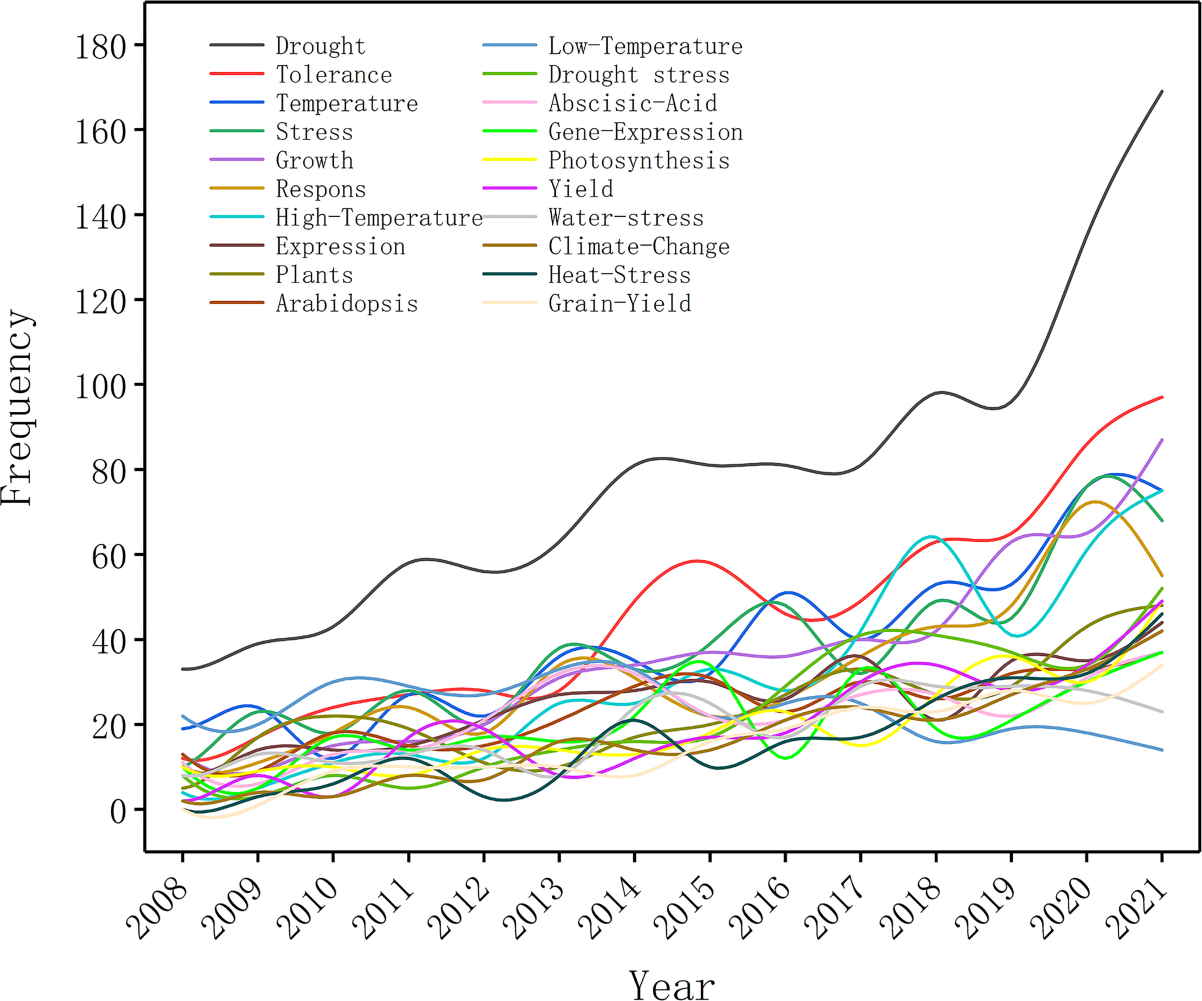
Trend of appearance frequency of the top 20 keywords in research papers on plant responses to high temperature and drought from 2008 to 2021.

Keywords indicate not only the research direction, but also changes in related research hot spots. In this study, we screened the top 20 commonly frequent keywords for plant responses to high temperature and drought from 2008 to 2021 (**Figure 5**). During 2008-2012, the frequencies of all keywords were relatively low, indicating that research on plant responses to high temperature and drought was still in infancy (**Figure 5**). Keywords and their frequencies have changed over time. The keywords of “drought”, “abiotic stress”, “drought stress”, “stress”, and “photosynthesis” were the top 5 high-frequency keywords during 2008-2012 (**Figure 5**), which showed that drought stress greatly influenced plant photosynthesis, and the changes in plant photosynthesis under abiotic stress have become hot spots during this period (**Table 6**). The keywords of “climate change”, “heat stress” and “drought” frequency increased rapidly from 2013-2017 and maintained the high frequency during 2018-2021. Given that these factors (heat stress, drought and climate change) greatly affected the growth and development of plants, researchers were keeping a high attention on them (**Table 6**). During 2013-2017 and 2018-2021, the top 5 keywords were “drought”, “heat stress”, “abiotic stress”, “climate change”, and “drought stress”. Therefore, research on the impacts of abiotic stress on plants under climate change, particularly high temperature stress and drought stress, has always been a social hotspot over the last 10 years. In general, research into plant responses to high temperatures and drought has been advanced rapidly during these two periods, with an increasing focus on future climate change.

**Table 6:** Dynamic changes of the top 20 keywords in the studies of plant responses to high temperature and drought during 2008-2012, 2013-2017, and 2018-2021.

Rank	Years					
	2008-2012		2013-2017		2018-2021	
	keyword	times	keyword	times	keyword	times
1	Drought	133	Drought	235	Drought	218
2	Abiotic stress	88	Abiotic stress	153	Heat stress	157
3	Drought stress	55	Heat stress	133	Climate change	153
4	Stress	51	Climate change	119	Abiotic stress	152
5	Photosynthesis	47	Drought stress	106	Drought stress	103
6	Climate change	41	Photosynthesis	90	Salinity	35
7	Heat stress	41	Wheat	69	Climate	21
8	Temperature	40	Water stress	58	Growth	21
9	Water stress	30	High temperature	56	Phytohormones	20
10	Salt stress	21	Gene expression	46	*Arabidopsis*	20
11	Chlorophyll fluorescence	19	Drought tolerance	42	*Triticum aestivum*	20
12	Transcription factor	18	Heat	41	Chlorophyll	20
13	Water	18	Stomatal conductance	27	Stomatal conductance	17
14	Salinity	16	Oxidative stress	24	Proline	17
15	Growth	14	Grain yield	22	Agriculture	12
16	Transgenic plants	13	Aba	15	Climate variability	6
17	Salt	10	Osmotic stress	14	Index	6
18	Transgenic tobacco	9	Climate	14	Photosystem ii	6
19	Phenotyping	7	Antioxidant	11	Relative water content	6
20	Remote sensing	7	Chlorophyll	10	Brassica	5

**Figure 6:**
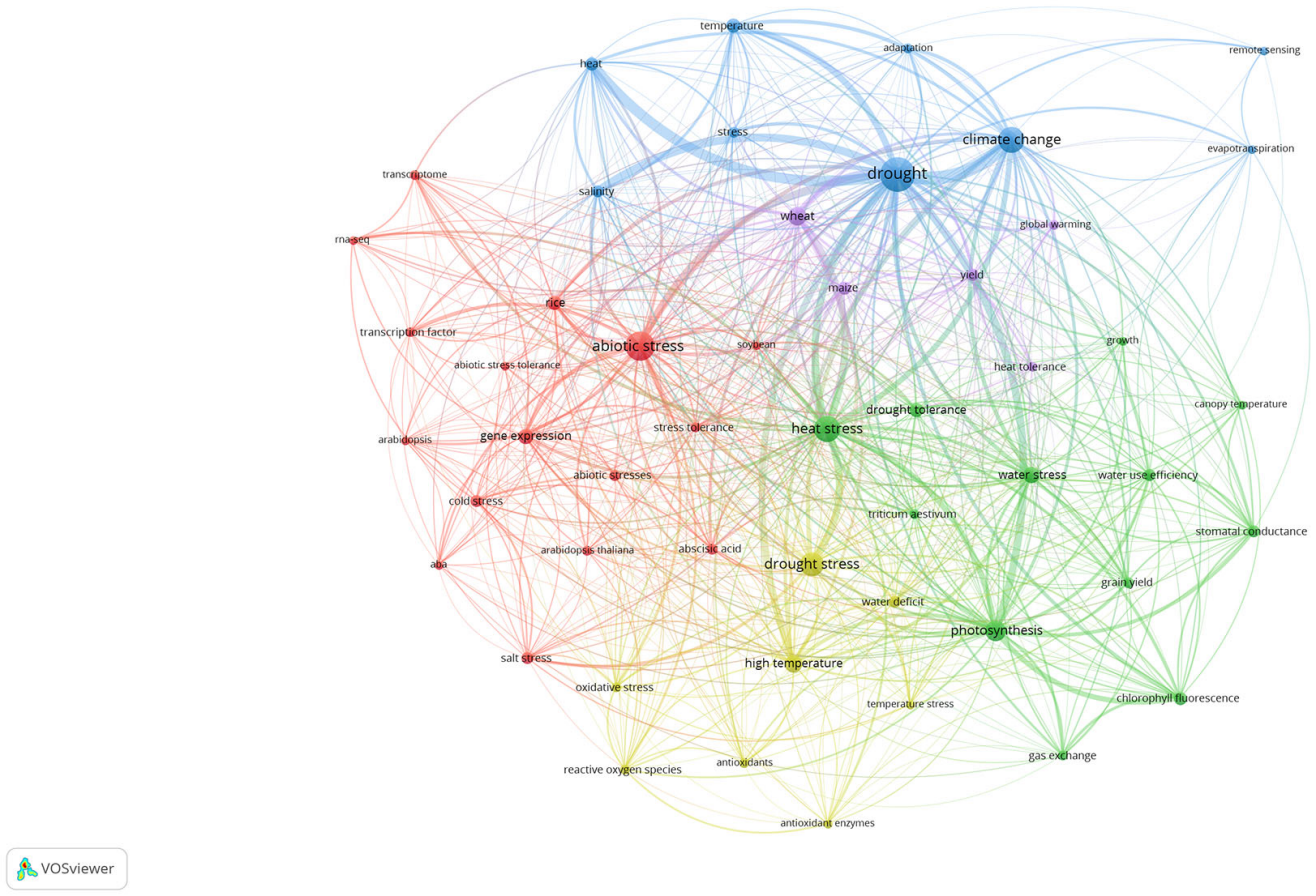
Co-occurrence network analysis of the top 50 keywords based on the VOS clustering algorithm. Different colored areas represent different clustering results. The red, blue, green, yellow, and purple connecting lines indicate the co-occurrence of keywords. The size of each node represents the frequency of the keyword appearing in the article.

We used the VOS algorithm to cluster the top 50 keywords related to research on plant responses to high temperature and drought from 2008 to 2021. All keywords were clustered into 5 types in the co-occurrence network (**Figure 6**). There were 16 keywords in the red cluster, mainly including abiotic stress, gene expression, stress tolerance, *Arabidopsis*, soybean. Researchers regarded *Arabidopsis thaliana*, which has a short growth cycle and a small genome, as a model plant. Based on the cluster analysis, scientific experiments were carried out on *Arabidopsis thaliana* with various stress treatments, studying its gene expression and plant stress resistance to improve grain yield in some extreme climate regions. Thus, the red cluster’s main research focus was on *Arabidopsis thaliana* molecular biology under abiotic stress, such as drought and high temperature stress. The green cluster has 12 keywords (**Figure 6**), mainly including photosynthesis, heat stress, drought tolerance, gas exchange and growth. Based on the cluster analysis, photosynthesis became the main research goal, and researchers shifted their focus to plant physiology and ecology. Meanwhile, they paid more attention to the physiological processes, such as gas exchange, water use efficiency, chlorophyll fluorescence, stomatal conductance. There were 9 keywords in the blue cluster: drought, climate change, heat, stress, adaptation, etc. The clustering is concentrated in the context of global warming, and how plants respond to high temperature stress and drought stress. The yellow cluster had a total of 8 keywords, which mainly were high temperature, drought stress, antioxidant enzymes, oxidative stress and reactive oxygen species. Cluster analysis found that researchers mainly focused on the biochemical reactions of plants under high temperature stress and drought stress. Finally, the purple cluster had only 5 keywords of global warming, wheat, maize, yield, and heat tolerance. The cluster analysis shows that high temperature, drought, and other abiotic stresses had great impact on food crops, The occurrence frequency of keywords, such as wheat and maize, has increased, implying that future climate change characterized with high temperature and drought will be likely to affect the yield of major food crops such as wheat and maize.

## Discussion

Although the current magnitude of global warming is still relatively low, some areas of the world have observed the phenomenon of tree death caused by abiotic stresses such as high temperature stress and drought stress (Allen et al., 2010). To face the potential severe climatic conditions in this century, more research on plant responses to high temperatures and drought is required. It is well known that plants often have trade-offs between stress resistance and high yield, thus resolving this conflict has always been one of the hot spots of research in this field. However, Jeong et al. (2010) determined that OsNAC10 can improve the stress resistance of plants and increase food production. It is exciting that the research on plant responses to high temperature and drought has attracted more and more attention from researchers in different countries, especially in the past 10 years (**Figure 1A**). According to journal papers analysis, fields of research related to plant responses to high temperature and drought mainly included phytology, agronomy, environmental science, biochemistry and molecular biology, and forestry. In addition, the journals of Frontiers in Plant Science (the highest number of total citations and total papers among all journals) (**Table 1**) and Journal of Experimental Botany (the second highest number of total citations among all journals) (**Table 1**) had much higher total citations than other journals in the research of plant responses to high temperature and drought. Despite that researchers have made many original and significant contributions to plant responses to high temperature and drought, more in-depth research in this field is needed. Moreover, the research on cell biology and molecular biology in this field has attracted extensive attention from researchers, which is demonstrated by collaborations between institutions and highly cited authors (**Figure 4** and **Table 2**).

From the top 20 most cited papers and the most cited references (**Tables 4** and **Table 5**), these studies focused on the yield of individual plants and the response of genes to high temperature and drought stress. As high temperature stress and drought stress limit agroforestry production, researchers have conducted in-depth research on this topic and made some progress. Rizhsky et al. (2002) combined cDNA arrays with physiological measurements to investigate the effects of drought and heat shock on tobacco (*Nicotiana tabacum*) plants. Yang et al. (2018) provided an overview of the environmental factors that contributed to fluctuations in plant secondary metabolite SMs. Fahad et al. (2017) generated a comprehensive overview of traditional and modern approaches to deal with heat and drought stress. Hundertmark and Hincha (2008) conducted a genome-wide analysis of *Arabidopsis thaliana* lea protein and its encoding genes. Jeong et al. (2010) reported a functional genomic approach for the identification of the regional gene OsNAC10, which led to a significant increase in grain yield in the field under drought conditions. Besides, Allen et al. (2010) made the first global assessment in about three decades of word wide tree mortality resulted from drought and high temperature stress. These advances have provided ideas of research in this field over the last 14 years, which makes a great contribution to plant production.

We found that research trends were related to practical problems faced at different time periods. Yet, what remains unchanged was that most papers focused on improving crop yields and vegetation stress tolerance, among which genomics research may be one of the solutions for agroforestry to cope with high temperature and drought stress in some regions in the future. It is well known that plants absorb water and nutrients through root system (Liu et al., 2017), thus rhizosphere microorganisms and root exudates may affect plant growth and development as well as strategies for stress tolerance (Huang et al., 2014). Despite the importance of plant root components, it is less examined in the highly cited paper, which mainly focused on the aboveground part of plants. However, Dinneny et al. (2008) studied the transcriptional response of *Arabidopsis thaliana* at different root cell layers to abiotic stresses across developmental stages. Besides, Jeong et al. (2010) reported a functional genomics approach to identify the domain gene OsNAC10. Nonetheless, these studies were concerned with the genomics and transcriptomics under single stress. Less attention has been paid to root morphology, rhizosphere microorganisms and root exudates under the combined stress of high temperature and drought. Future research is needed to enhance the understanding of plant responses to high temperature and drought by linking plant belowground components.

The topic of research generally focused on the problems that demanded solutions or the implementation of national policies. The evolution of keywords at various times can be used as the bellwether of research topics. Between 2008 and 2012, the frequency of these keywords was usually low due to the small number of papers. “Drought” and “abiotic stress” were the two keywords with the highest frequency (**Table 6**). This means that drought was an early focus for researchers, who aimed to improve grain yields and minimize the impacts of drought stress on agriculture in arid regions (Jeong et al., 2010). From 2013 to 2021, the frequency of occurrence of all keywords increased over the past 10 years. “Drought”, “abiotic stress” “heat stress” and “climate change” had the highest frequency, with “heat stress” and “climate change” increased more rapidly. The melting of Arctic glaciers as well as forest mortality under extreme droughts and high temperatures in local areas, has prompted researchers to focus on global climate change (Allen et al., 2010; Allan et al., 2021). Numerous studies have shown that vegetation growing seasons in the mid-to-high latitudes of the Northern Hemisphere were prolonged due to global warming (Rice et al., 2018; Jeong et al., 2011; Liu et al., 2016; Liu et al., 2021). Therefore, it is of great significance to study the impacts of high temperature and drought caused by future climate change on plant growth and development.

After understanding the past and current goals of plant responses to high temperature and drought research, it is necessary to explore trends for future research. In the cluster analysis of the top 50 keywords, the main research direction of the red cluster is the molecular biology research of *Arabidopsis thaliana* under abiotic stress, such as high temperature stress and drought stress (Zhang et al., 2010; Zafar et al., 2020). In contrast, the green cluster mainly focused on plant physiology, such as gas exchange and water use strategies (Letts et al., 2009; Belko et al., 2013; Elferjani and Soolanayakanahally, 2018). Both clusters were the main research directions for the period 2008-2021, and will continue to be the research focus in the future. The blue cluster mainly focused on that in the context of global climate change, it was of growing interest to investigating how plants adapt to high temperature stress and drought stress (Wang et al., 2014; Steward et al., 2018). The yellow cluster analysis found that researchers mainly focused on plant biochemistry under high temperature stress and drought stress (Huang et al., 2012; Hong et al., 2013; Xia et al., 2014) (**Figure 6**). Research in these two clusters grew steadily from 2008 to 2021. In contrast, the purple cluster only included 5 keywords: global warming, wheat, maize, yield, and heat tolerance. Except heat tolerance, the other 4 keywords have just increased in frequency over the past 5 years (Devasirvatham and Tan, 2018; Hlavacova et al., 2018; El Sabagh et al., 2019; Mdluli et al., 2020). These increasing keywords may be the weather vane in the future. Atmospheric carbon dioxide is projected to rise up to 800 ppm by the end of the century and may exacerbate the negative effects of high temperature as well as drought stress on food crop yields (van Vuuren et al., 2011; Vermeulen et al., 2012), which will have substantial impacts on agriculture safety for increasing human population. Consequently, under future climate change, how to regulate the growth and development of food crops subjected to high temperature and drought stress may be a hot topic, and is of major importance for the future of global agricultural production.

## Conclusion

In this study, we used bibliometric methods to comprehensively and quantitatively evaluate the focus and trend of global research on plant responses to high temperature and drought. The “Advanced Search” of “Web of Science” (WOS) based on the “Web of Science Core Collections” database was used to screen out a total of 3782 papers, including 3343 articles, 318 review papers, 101 conference proceedings, and 13 book chapters, 4 editorial materials, 2 letters, and 1 retracted publication. Then we used the “bibliometrix” package in the R language and VOSviewer software to analyze all papers about research on plant responses to high temperature and drought. The main conclusions were drawn as follows.

(1) The number of papers published on the aspect of plant responses to high temperature and drought increased steadily from 2008 to 2014 (1199 papers) and then increased rapidly from 2015 to 2021 (2583 papers), indicating the future of research on plant responses to high temperature and drought is the hot spot.
(2) The main research areas of plant responses to high temperature and drought were summarized, including plant science, agronomy, environmental science, biochemistry and molecular biology, and forestry. The research papers involved have been published in various plant-related journals, with the most published in journal of Frontiers in Plant Science, which had the highest global total citations and H-index. By contrast, the one with the highest number of local total citations was the journal of Plant Physiology.
(3) For the contributions of different countries, institutions, and individuals to the research on plant responses to high temperature and drought, the number of papers and total citations in China, USA and India has always maintained a high level during 2008-2012, 2013-2017, and 2018-2021. Based on different research characteristics and directions, the cooperation between institutions was mainly represented by Northwest A&F University, Nanjing Agricultural University, and China Agricultural University. For authors, S. V. Krishna Jagadish, Xinxin Zhang, and Ashraf M were the most influential and competitive authors of plant responses to high temperature and drought research.
(4) According to the summaries of the most cited papers and references, the most crucial research hotspots of plant responses to high temperature and drought were the effects of drought stress on plant yields, the growth and development of plants under abiotic stress, and the genomics research.
(5) Keywords of “drought”, “heat stress”, “climate change”, and “abiotic stress” were the focuses of plant responses to high temperature and drought over the past 10 years. This is demonstrated from the grouping results of the 50 main keywords for studies on plant responses to high temperature and drought. Cluster analysis of keywords shows that high temperature, drought, and other abiotic stresses had great impacts to food crops. The occurrence frequency of keywords such as wheat and maize have increased and implies that future climate change characterized with high temperature and drought will be likely to affect the yield of major food crops such as wheat and maize.
(6) Under future climate change, how to regulate the growth and development of food crops subjected to high temperature and drought stress may become a hotspot, and is of major importance for the future of global agricultural safety. In addition, less studies have been conducted on root morphology, rhizosphere microorganisms and root exudates under the combined stress of high temperature and drought. Thus, increasing research is critical to provide more insights into plant responses to high temperature and drought by linking plant above-below ground components, as the climate is likely to become hotter and drier. In general, this study systematically reveals the development, trend, and prospects of plant responses to high temperature and drought research, which can deepen our understanding of how plants cope with future climate change.

## Data availability statement

The original contributions presented in the study are included in the article/supplementary material. Further inquiries can be directed to the corresponding author.

## Author contributions

HD conceived this study. YC, YZ, and CS conducted the data collection and analysis. YC wrote the manuscript with input from SO, LT, and HD. All authors contributed to the article and approved the submitted version.

## Funding

This work was supported by the Natural Science Talent Funding of Guizhou University (202132).

## Conflict of interest

The authors declare that the research was conducted in the absence of any commercial or financial relationships that could be construed as a potential conflict of interest.

## Publisher’s note

All claims expressed in this article are solely those of the authors and do not necessarily represent those of their affiliated organizations, or those of the publisher, the editors and the reviewers. Any product that may be evaluated in this article, or claim that may be made by its manufacturer, is not guaranteed or endorsed by the publisher.
